# Measuring Bacterial Growth Potential of Ultra-Low Nutrient Drinking Water Produced by Reverse Osmosis: Effect of Sample Pre-treatment and Bacterial Inoculum

**DOI:** 10.3389/fmicb.2020.00791

**Published:** 2020-04-29

**Authors:** Mohaned Sousi, Sergio G. Salinas-Rodriguez, Gang Liu, Jan C. Schippers, Maria D. Kennedy, Walter van der Meer

**Affiliations:** ^1^Department of Environmental Engineering and Water Technology, IHE Delft Institute for Water Education, Delft, Netherlands; ^2^Faculty of Science and Technology, University of Twente, Enschede, Netherlands; ^3^Key Laboratory of Drinking Water Science and Technology, Research Centre for Eco-Environmental Sciences, Chinese Academy of Sciences, Beijing, China; ^4^Department of Water Management, Faculty of Civil Engineering and Geoscience, Delft University of Technology, Delft, Netherlands; ^5^Oasen Drinkwater, Gouda, Netherlands

**Keywords:** reverse osmosis, remineralisation, ultra-low nutrient water, bacterial growth potential, pre-treatment, bacterial inoculum

## Abstract

Measuring bacterial growth potential (BGP) involves sample pre-treatment and inoculation, both of which may introduce contaminants in ultra-low nutrient water (e.g., remineralized RO permeate). Pasteurization pre-treatment may lead to denaturing of nutrients, and membrane filtration may leach/remove nutrients into/from water samples. Inoculating remineralized RO permeate samples with natural bacteria from conventional drinking water leads to undesired nutrient addition, which could be avoided by using the remineralized RO permeate itself as inoculum. Therefore, this study examined the effect of pasteurization and membrane filtration on the BGP of remineralized RO permeate. In addition, the possibility of using bacteria from remineralized RO permeate as inoculum was investigated by evaluating their ability to utilize organic carbon that is readily available (acetate, glucose) or complex (laminarin, gelatin, and natural dissolved organic carbon), as compared with bacteria from conventional drinking water. The results showed that membrane filtration pre-treatment increased (140–320%) the BGP of remineralized RO permeate despite the extensive soaking and flushing of filters (>350 h), whereas no effect was observed on the BGP of conventional drinking water owing to its high nutrient content. Pasteurization pre-treatment had insignificant effects on the BGP of both water types. Remineralized RO permeate bacteria showed limitations in utilizing complex organic carbon compared with bacteria from conventional drinking water. In conclusion, the BGP bioassay for ultra-low nutrient water (e.g., remineralized RO permeate) should consider pasteurization pre-treatment. However, an inoculum comprising bacteria from remineralized RO permeate is not recommended as the bacterial consortium was shown to be limited in terms of the compounds they could utilize for growth.

## Introduction

Bacterial growth in water supply systems, whether in the form of planktonic bacteria or biofilms attached to surfaces in contact with water, is associated with health threats [e.g., diseases caused by pathogenic bacteria, such as Legionella pneumophila (Prest et al., 2016b)], operational problems of water supply (e.g., bio-corrosion of pipe material), and adverse effects on the esthetic characteristics of drinking water (Volk and LeChevallier, 1999; Berry et al., 2006; Liu et al., 2017). Controlling bacterial growth during water distribution, especially in non-chlorinated systems, requires the production of biologically stable drinking water with a very low concentration of biodegradable organic compounds that provide energy for bacterial bioprocesses and proliferation (Prest et al., 2016b; Nescerecka et al., 2018). Reverse osmosis (RO) filtration is capable of producing drinking water with ultra-low nutrient level, and thus, very low bacterial growth potential (BGP) (Park and Hu, 2010; Dixon et al., 2012). However, Sousi et al. (2018) demonstrated that the existing BGP bioassays are not suitable for ultra-low nutrient water, such as remineralized RO permeate (BGP = 50–100 × 10^3^ cells/mL), because of their high detection limit. Additionally, more care should be taken when measuring the BGP of ultra-low nutrient water samples due to the high susceptibility of this water type to sample preparation procedures that can introduce contamination (i.e., BGP increase). There are several methodological aspects that can affect obtaining reliable BGP results, including glassware preparation, surrounding laboratory environment, chemicals addition, sample pre-treatment, and inoculation. This study, however, focused on the last two aspects (i.e., sample pre-treatment and inoculation) due to their potentially high effect, and the variation of sample pre-treatment methods and inoculum types proposed in the literature as explained below.

Sample pre-treatment is performed to inactivate or remove indigenous bacteria, where membrane filtration (0.1- or 0.2-μm) (Servais et al., 1987; Percherancier et al., 1996; Hammes and Egli, 2005), pasteurization (60 or 70°C) (van der Kooij et al., 1982; Joret et al., 1991; van der Kooij, 1992; Sack et al., 2010, 2011), or a combination of these two methods (Park et al., 2016) are used. Alternatively, some bioassays consider no sample pre-treatment (i.e., direct incubation) (Prest et al., 2016a), arguing that it may affect the nutrient nature in water (Ross et al., 2013). However, Sousi et al. (2018) demonstrated that pre-treatment is needed for ultra-low nutrient water samples in which initial cell count exceeded the available nutrients for bacterial growth (e.g., wash-out of bacterial loads from filtration units), leading to a decreasing number of cells over time directly after starting the bioassay. The main disadvantage of pasteurization is that it may affect the nature of organic compounds in water, whereas filtration may either remove or leach nutrients from/into water (Khan and Subramania-Pillai, 2006), resulting in unreliable BGP outcome. Although researchers in this field are aware of these potential drawbacks (Ross et al., 2013), no explicit studies have been conducted to assess the effects of sample pre-treatment on the BGP outcome.

Inoculating ultra-low nutrient water samples after pre-treatment with natural bacteria is essential to initiate growth, where undesired nutrient addition can occur when using nutrient-rich water as an inoculum source (Sousi et al., 2018). Instead, ultra-low nutrient water (e.g., remineralized RO permeate) can be used as an inoculum source to reduce the undesired nutrient addition, especially that (remineralized) RO permeate bacteria are best adapted to grow in their own water without the need for an external inoculum as demonstrated by Sousi et al. (2018), which was also observed for other water types (Farhat et al., 2018). However, investigating the ability of (remineralized) RO permeate bacteria to utilize organic carbon of various complexity levels is first needed, especially with the considerably low diversity of the RO permeate bacterial species (Belila et al., 2016).

The BGP bioassay based on cell count is considered for this study, where the results can be expressed as the maximum cell count obtained during the incubation period (Sousi et al., 2018), or as the net bacterial growth which is the difference between the initial and maximum cell counts as in the direct incubation methods (Prest et al., 2016a; Nescerecka et al., 2018).

The objective of this study was, therefore, to assess the effect of sample pre-treatment by pasteurization and membrane filtration on the BGP (i.e., maximum and net bacterial growth) of ultra-low nutrient drinking water produced by RO filtration and remineralization. In addition, the possibility of using bacteria naturally present in remineralized RO permeate as inoculum for BGP measurements was investigated by testing their ability to utilize organic carbon with various molecular characteristics.

## Materials and Methods

### Water Samples

This study was conducted on the Oasen’s drinking water treatment plant (Kamerik, Netherlands), which produces 340 m^3^/h of drinking water by conventional treatment of anaerobic groundwater. The treatment consists of dry sand filtration, pellet softening, rapid sand filtration, activated carbon filtration (15 min), and medium-pressure ultraviolet disinfection (20 mJ/cm^2^). For research purposes, anaerobic groundwater is also treated by a pilot-scale advanced treatment unit (7 m^3^/h) comprising anaerobic RO filtration (75% total recovery), followed by post-treatment processes: ion exchange, remineralization using calcite contactors, and tower aeration. Finished waters of both treatment lines, i.e., conventionally treated water after the clean water reservoir (CTW) and site-remineralized RO permeate after all post-treatment processes (site-Remin), were sampled for BGP measurements. Water properties of CTW and site-Remin are given in Supplementary Table S1. In addition, water collected directly after RO filtration (i.e., RO permeate) was used as a blank for BGP measurements as described in the following section. Lastly, anaerobic groundwater (AGW) and activated carbon filtrate (ACF) were used as sources for natural bacterial inoculum. Samples were collected on a monthly basis in the period between October 2016 and December 2018.

### Bacterial Growth Potential Bioassay

The BGP bioassay proposed by Sousi et al. (2018) was applied in this study, in short:

Sample collection: water samples were collected in AOC-free glassware, which have been muffled at 550°C for 6 h (Prest et al., 2016a).

Sample handling: water samples were pre-treated to inactivate indigenous bacteria prior to the addition of a natural bacterial inoculum. The full pre-treatment and inoculation details are given in the following section. Thereafter, each water sample was divided into three AOC-free glass vials, which were incubated in the dark at 30°C for 20 days.

Flow cytometry (FCM): cell count in the incubated water samples was measured (more frequently in the first week) using BD Accuri C6^®^ FCM (BD Biosciences, Belgium) as described by Prest et al. (2016a), where only intact cell count was reported as the increase in total cell count was mainly due to the new intact cells formed (Supplementary Figure S1). Intact cells were stained with a mix of SYBR Green I (1:100) and propidium iodide (0.3 mM PI), where 5 μL stain was added to 500 μL of pre-heated samples at 35 ± 2°C. Thereafter, samples were post-heated at the same temperature for 10 min before conducting the FCM measurements.

The blank: The BGP blank was prepared by remineralizing RO permeate at the laboratory with 122 mg/L HCO_3_^–^ (final pH of 7.8 ± 0.2), 40 mg/L Ca^2+^, 4 mg/L Mg^2+^, 5 μg-P/L, and 50 μg-N/L. The blank (laboratory-remineralized RO permeate) is denoted as lab-Remin and has a BGP of 50 ± 20 × 10^3^ intact cells/mL.

### Experimental Approach

The following methodological aspects were investigated:

(i) The effect of sample pre-treatment was studied by measuring the BGP of lab-Remin, site-Remin, and CTW samples with different pre-treatments: pasteurization at 70°C for 30 min using a water bath (type 1008 Water Bath, GFL, Germany), autoclaving (3870 ELV autoclave, Tuttnauer Europe, Netherlands) at 121°C for 15 min, and membrane filtration using gamma-sterilized polyethersulfone filters (0.22-μm pore size, 33-mm diameter, Merck Millipore Ltd., Ireland). Pre-treatment by pasteurization or autoclaving was carried out on 200 mL of water sample contained in 250 mL Duran glassware, where the effective heating time was calculated after the water samples reached the required temperature. Thereafter, samples were allowed to cool to room temperature (∼20°C) in an ice bath before any further handling. Moreover, pasteurization for a longer duration (45, 60, 90, and 120 min) was carried out on CTW samples to investigate the effect of heat duration on denaturing of nutrients. Regarding membrane filtration, all filters and syringes were thoroughly cleaned before using by soaking for 360 h in ultra-pure water (Milli-Q^®^ water, Merck Millipore) interspersed with daily flushing (200 mL Milli-Q water) to avoid leaching of organic carbon in the samples. Water samples were filtered in 250 mL Duran glassware before distributing in triplicate vials. Additionally, BGP of non–pre-treated samples was measured as a control. The potential denaturing of nutrients due to sample pre-treatment was studied by analyzing the water samples before and after pre-treatment for: AOC (P17/NOX) (van der Kooij and Hijnen, 1984), dissolved organic carbon (DOC) (Shimadzu TOC-L, Japan), and liquid chromatography–organic carbon detection (LC–OCD) (Huber et al., 2011) to measure the biodegradable fractions of DOC (e.g., biopolymers and humic substances).
(ii) The ability of (remineralized) RO permeate bacteria to utilize readily available (glucose and acetate) and complex (laminarin, gelatin, and natural DOC) organic compounds was tested. The bacterial yield (based on BGP at 0, 50, and 500 μg-C/L final concentration in the sample) was calculated for lab-Remin (the blank) without inoculation (i.e., only indigenous bacteria of RO permeate are present, <10^3^ intact cells/mL with FCM) and with the addition of four different natural bacterial inocula: site-Remin, CTW, ACF, or AGW. The final inoculum concentration in the water sample was in the range of 2–10 × 10^3^ intact cells/mL (1.7 ± 0.2%, v/v), depending on the initial cell count of the inoculum source, which was in average: 25 × 10^3^, 600 × 10^3^, 500 × 10^3^, and 290 × 10^3^ intact cells/mL, for site-Remin, CTW, ACF, and AGW, respectively. Stock solutions (1,000 ± 50 mg-C/L) of sodium acetate, glucose, laminarin (from Laminaria digitata), and gelatin (type B, from bovine skin) were prepared using Milli-Q water in AOC-free bottles and kept at 4°C. Additionally, RO concentrate was used as a source of natural DOC (∼33 mg-C/L), where it was filtrated (0.45-μm pore size, PVDF, 33-mm Ø, Merck Millipore Ltd., Ireland), pasteurized (70°C for 30 min), and then filtrated again (0.45-μm) to ensure complete removal of suspended particles and bacteria. The treated RO concentrate was added to lab-Remin at a ratio of ∼8% to obtain a final DOC concentration of 2.7 mg-C/L. Phosphorus (5 μg-P/L) and nitrogen (50 μg-C/L)were added to all the samples, regardless of the added carbon concentration, to ensure that carbon was the growth-limiting factor during the test.

**FIGURE 1:**
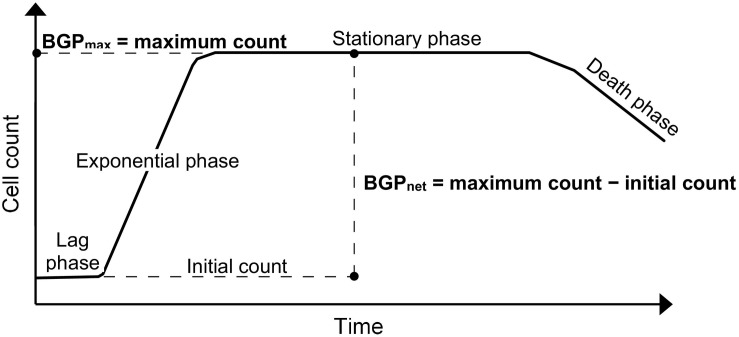
Typical bacterial growth curve illustrating the different ways of expressing bacterial growth potential (BGP) results: maximum bacterial growth(BGP_*max*_) and net bacterial growth (BGP_*net*_).

Lastly, BGP results can be expressed as maximum bacterial growth (BGP_max_) or net bacterial growth (BGP_net_) as illustrated in Figure 1. Both ways of expressing BGP results were assessed by measuring the BGP of pasteurized CTW after inoculation with three different concentrations of bacteria originating from the same water type: 10 × 10^3^, 100 × 10^3^, and 250 × 10^3^ intact cells/mL. The BGP of non–pre-treated and non-inoculated CTW was measured as a control.

### Statistical Analysis

Student’s t-test and one-way analysis of variance (ANOVA) were used to determine the significance of differences between samples with normally distributed data (affirmed by Q–Q plots, Chi-squared tests, and Kolmogorov-Smirnov tests). In addition, a simple linear correlation between two quantitative variables was applied. The Microsoft Excel software was used for statistical analysis with 95% confidence (alpha of 0.05).

## Results

### Expressing BGP Results

**FIGURE 2:**
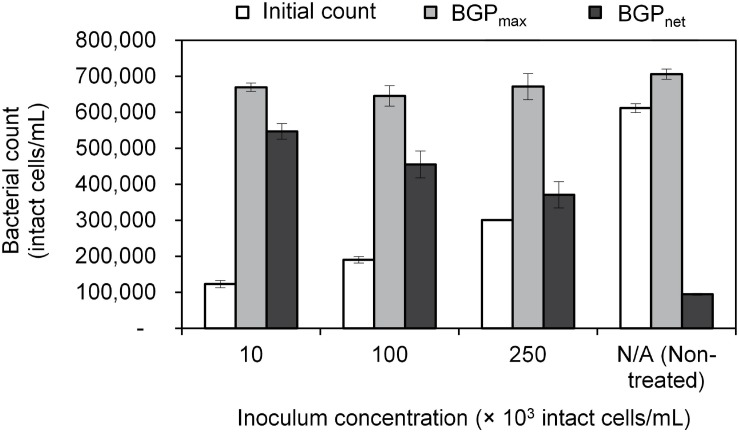
Initial cell count, maximum bacterial growth potential (BGP_max), and net bacterial growth potential (BGP_*net*) of pasteurized conventionally treated water with varying inoculum concentration (10 × 10^3^, 100 × 10^3^, and 250 × 10^3^ intact cells/mL) originating from the same water. Error bars represent the measurement variations of three separate tests, with triplicate vials per test.

The maximum bacterial growth (BGP_max_) of pasteurized and inoculated conventionally treated water (CTW) remained in the range of 650–700 × 10^3^ intact cells/mL regardless of the initial cell count which varied from 100–600 × 10^3^ intact cells/mL (Figure 2). As a result, the net bacterial growth (BGP_net_) varied significantly among the samples (P < 0.05). Interestingly, pasteurization (at 70°C for 30 min) was not sufficient to completely remove intact cells, where 100 × 10^3^ intact cells/mL were found in the pasteurized CTW before inoculation. Those pasteurization-resistant cells seemed to be inactive, as no bacterial growth was observed in the pasteurized but not inoculated CTW samples during the 20-day incubation period. However, a long term test showed that bacterial growth occurred at a very low rate, where the bacterial count reached 280 × 10^3^ intact cells/mL after about 80 days of incubation.

### Effect of Sample Pre-treatment on BGP

**FIGURE 3:**
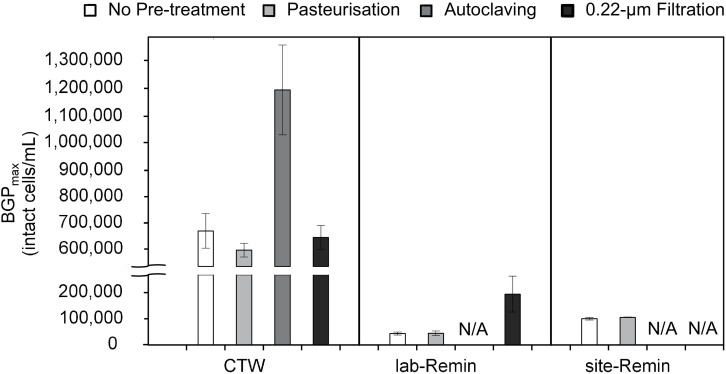
Maximum bacterial growth potential (BGP_max; 20 days at 30°C) of non–pre-treated, pasteurized (70°C for 30 min), sterilized (121°C for 15 min), and 0.22-μm filtrated water samples of lab-remineralized RO permeate (lab-Remin, the blank), site-remineralized RO permeate (site-Remin), and conventionally treated water (CTW). All samples were inoculated with CTW whether pre-treated or not. Error bars represent the measurement variations of three separate tests, with triplicate vials per test. N/A, not available.

The effect of sample pre-treatment on the BGP of water was highly dependent on the type of water. The results (Figure 3) revealed that the BGP_max_ of CTW was comparable (P > 0.05) whether the samples were non–pre-treated (677 ± 60 × 10^3^ intact cells/mL, the control) or pre-treated by pasteurization (610 ± 25 × 10^3^ intact cells/mL) or 0.22-μm filtration (655 ± 40 × 10^3^ intact cells/mL). Moreover, pasteurization of the CTW samples for a longer period (45, 60, 90, and 120 min) had minor influences on the BGP_max_ (Supplementary Figure S2). In contrast, the BGP_max_ of the autoclaved CTW samples increased by 75–85% (1,180 ± 160 × 10^3^ intact cells/mL). For ultra-low nutrient water (i.e., the lab-Remin blank and site-Remin), insignificant (P > 0.05) differences in BGP_max_ were observed between the pasteurized and non–pre-treated (the control) samples. The BGP_max_ of lab-Remin was 41 ± 8 × 10^3^ and 40 ± 5 × 10^3^ intact cells/mL for the pasteurized and non–pre-treated samples, respectively, and the corresponding BGP_max_ of site-Remin was 98 ± 2 × 10^3^ and 94 ± 5 × 10^3^ intact cells/mL. Unlike the results for CTW, 0.22-μm filtration had a substantial influence on the BGP_max_ of lab-Remin, which increased by 140–320% (to 180 ± 60 × 10^3^ intact cells/mL) in the samples pre-treated by filtration.

Notably, the AOC concentration in the samples (slightly) increased after any pre-treatment (Supplementary Table S2) including pasteurization which had minor effects on the BGP results. The increase in AOC concentration ranged from 1 (in lab-Remin and site-Remin) to 8 μg-C/L (in CTW) depending on the pre-treatment applied, and it was not strongly correlated with the increase in BGP_max_ (R^2^ = 0.40). Moreover, the concentrations of DOC and biodegradable carbon fractions measured by LC–OCD remained unchanged (Supplementary Table S2, DOC: 5.9 ± 0.1 mg-C/L for CTW, and <0.2 mg-C/L for site-Remin and lab-Remin) regardless of the pre-treatment applied.

### Assessing the Ability of (remineralized) RO Permeate Bacteria to Utilize Organic Carbon

**FIGURE 4:**
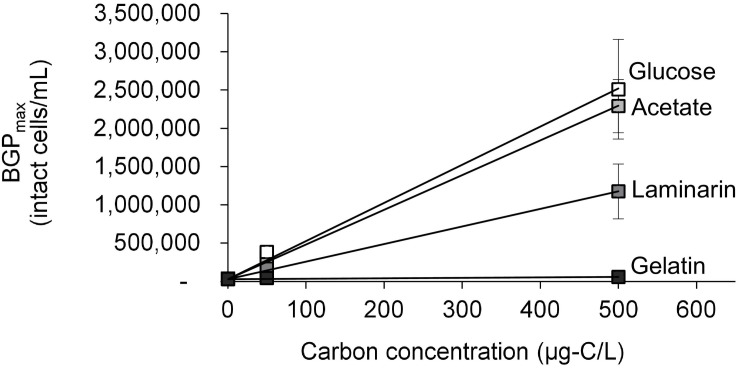
Maximum bacterial growth potential (BGP_max; 20 days at 30°C) of non-inoculated lab-remineralized RO permeate (lab-Remin) on different carbon sources. Error bars represent the measurement variations of three separate tests, with triplicate vials per test.

**FIGURE 5:**
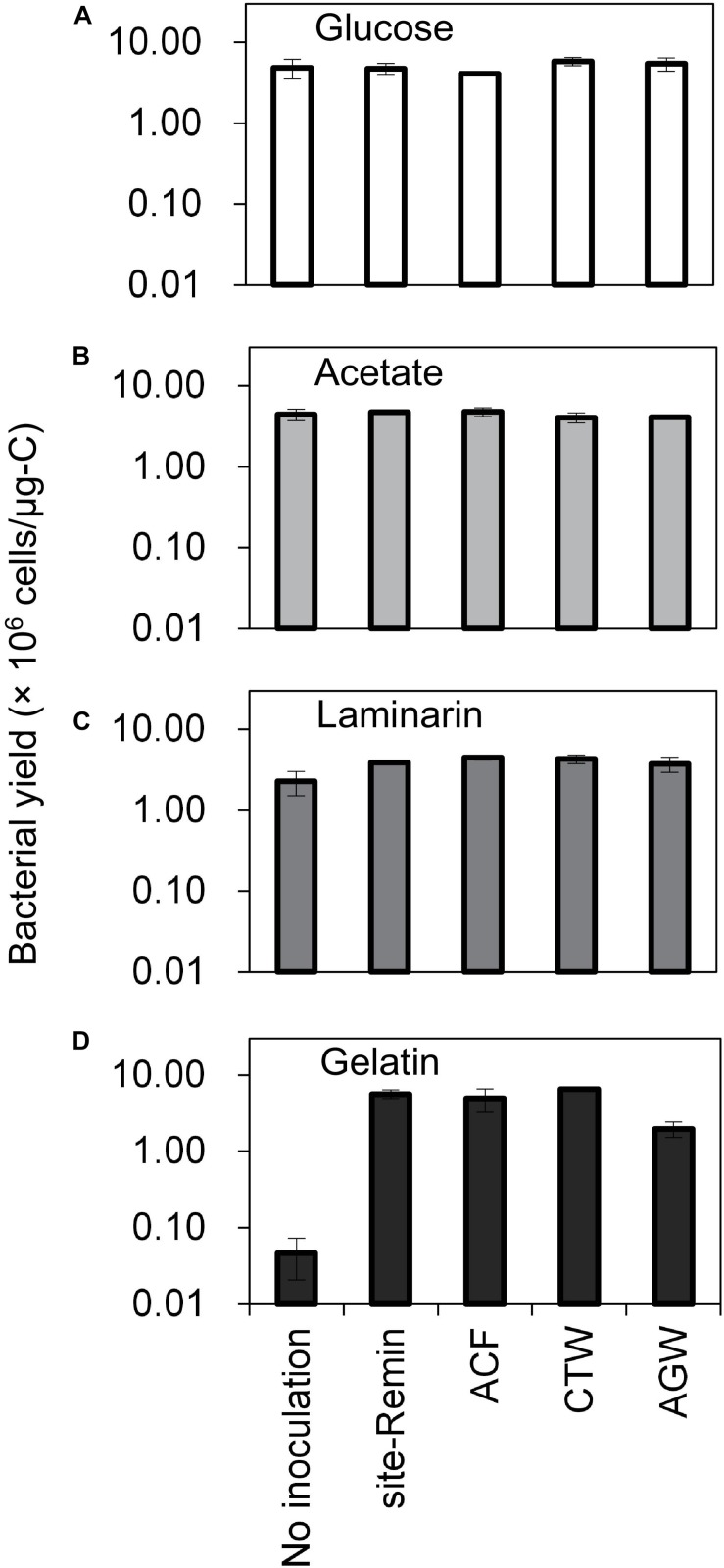
Bacterial yield in lab-remineralized RO permeate (lab-Remin) without inoculation or after inoculating with natural bacteria originating from: site-remineralized RO permeate (site-Remin), activated carbon filtrate (ACF), conventionally treated water (CTW), and anaerobic groundwater (AGW). Four sources of organic carbon were used: glucose **(A)**, acetate **(B)**, laminarin **(C)**, and gelatin **(D)**. Error bars represent the measurement variations of three separate tests, with triplicate vials per test.

**FIGURE 6:**
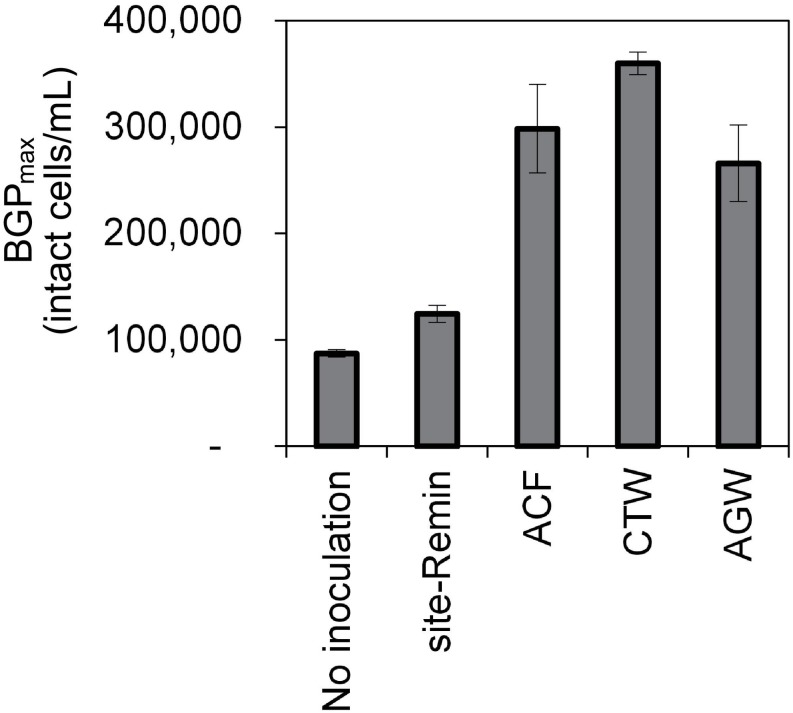
Maximum bacterial growth potential (BGP_max; 20 days at 30°C) of lab-remineralized RO permeate (lab-Remin) without inoculation or after inoculating with natural bacteria originating from: site-remineralized RO permeate (site-Remin), activated carbon filtrate (ACF), conventionally treated water (CTW), and anaerobic groundwater (AGW). The growth was measured after the addition of natural DOC (2.75 mg-C/L) originating from RO concentrate. Error bars represent the measurement variations of 3 separate tests, with triplicate vials per test.

The growth of natural bacterial consortia on organic carbon varied according to the source of bacteria as well as the type of organic carbon. The BGP_max_ of lab-Remin increased linearly with the addition of glucose and acetate, whether lab-Remin was not inoculated (i.e., only indigenous bacteria of RO permeate are present) or inoculated with different natural bacterial consortia (i.e., site-Remin, ACF, CTW, and AGW). Figure 4 shows the linear (R^2^ = 0.99) increase in BGP_max_ of the non-inoculated lab-Remin samples, and Supplementary Figure S3 shows the results of the inoculated ones. Moreover, the BGP_max_ was obtained within 2–5 days of incubation, reflecting the high biodegradability of glucose and acetate. The bacterial yield on glucose and acetate was comparable (P > 0.05) irrespective of the inoculum type, where the average yield was 5.00 ± 0.65 × 10^6^ cells/μg-C for glucose (Figure 5A) and 4.45 ± 0.35 × 10^6^ cells/μg-C for acetate (Figure 5B). On the other hand, the BGP_max_ on complex organic carbon (i.e., laminarin, gelatin, and natural DOC) was obtained within 10 to 15 days of incubation, and it was highly dependent on the inoculum type. For instance, the BGP_max_ obtained for non-inoculated lab-Remin was significantly (P < 0.05) lower than that obtained for inoculated lab-Remin. The growth in non-inoculated lab-Remin was partially limited when laminarin was used as the sole carbon source (Figure 5C), where the bacterial yield was 2.25 ± 0.75 × 10^6^ cells/μg-C compared with the average of 4.10 ± 0.35 × 10^6^ cells/μg-C observed for the other inocula combined. Moreover, the growth in non-inoculated lab-Remin was considerably low when gelatin was used as the sole carbon source (Figure 5D), where the bacterial yield was negligible (0.05 ± 0.04 × 10^6^ cells/μg-C). The yield in lab-Remin spiked with gelatin was also limited when AGW was used an inoculum (1.95 ± 0.45 × 10^6^ cells/μg-C) compared with site-Remin, ACF, and CTW inocula (5.70 ± 0.80 × 10^6^ cells/μg-C). Regarding the growth on natural DOC (2.75 mg-C/L) prepared from the pasteurized RO concentrate, the results revealed that the type of bacterial inoculum also significantly (P < 0.05) influenced the growth (Figure 6). The highest growth in lab-Remin was observed when natural bacterial inocula originating from CTW (350 ± 15 × 10^3^ intact cells/mL), ACF (300 ± 45 × 10^3^ intact cells/mL), and AGW (270 ± 35 × 10^3^ intact cells/mL) were used. Lower growth was observed with site-Remin inoculum (124 ± 8 × 10^3^ intact cells/mL), and the indigenous bacteria of RO permeate (i.e., no inoculation, 87 ± 4 × 10^3^ intact cells/mL).

### Monitoring the BGP of Conventionally Treated and RO-Treated Drinking Water

**FIGURE 7:**
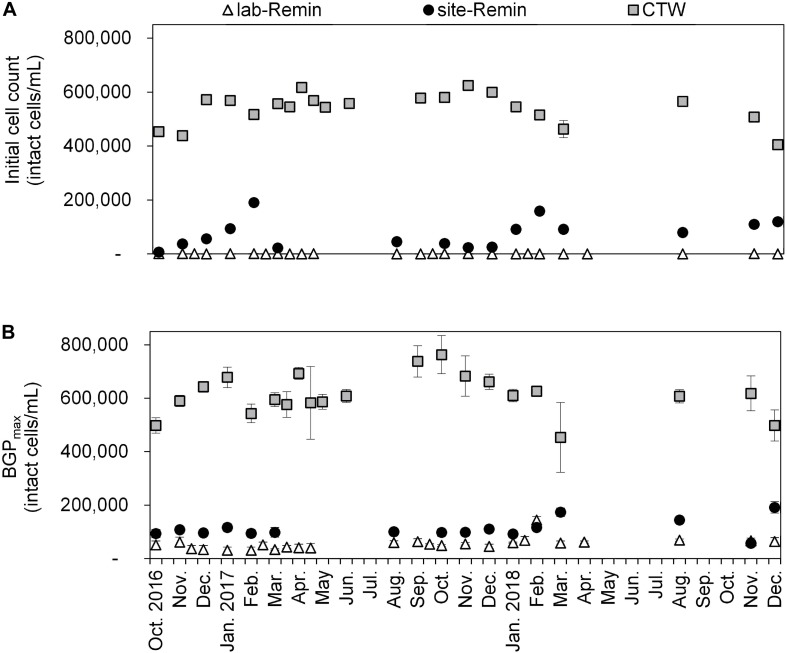
Initial intact cell count **(A)** and maximum bacterial growth potential (BGP_max, **(B)** of lab-remineralized RO permeate (lab-Remin, the blank), site-remineralized RO permeate (site-Remin), and conventionally treated water (CTW). All samples were pasteurized (70°C for 30 min) and inoculated with CTW. Error bars represent the variations of triplicate measurements.

The initial intact cell count and BGP of lab-Remin, site-Remin, and CTW were monitored for a period of 2 years (Figure 7). The results demonstrated superior performance of the RO-based treatment line, where the initial cell count of lab-Remin (<10^3^ intact cells/mL) and site-Remin (25–200 × 10^3^ intact cells/mL) were systematically lower than that of CTW (400–600 × 10^3^ intact cells/mL). Similarly, the BGP was subsequently reduced by >75% with the RO-based treatment line compared with the conventional one, where no pronounced seasonal variations were observed and the BGP was stable around 35–60 × 10^3^, 90–150 × 10^3^, and 500–700 × 10^3^ intact cells/mL for lab-Remin, site-Remin, and CTW, respectively.

## Discussion

This study focused on: assessing the effect of sample pre-treatment on the BGP of ultra-low nutrient water, and examining the ability of (remineralized) RO permeate bacteria to utilize readily available as well as complex organic carbon. Expressing the BGP results in the most appropriate way is essential to accurately interpret the test outcome, allowing for achieving the objectives of this study. Currently, the BGP results are expressed either as BGP_net_ for pre-treated (Vital et al., 2010; Park et al., 2016) and non–pre-treated (i.e., direct incubation) (Prest et al., 2016a; Nescerecka et al., 2018) samples, or as BGP_max_ (Joret et al., 1991; Percherancier et al., 1996; Sousi et al., 2018). The results of the present study suggest that BGP_max_ gives a better representation of the BGP of water, since the effect of initial cell count is ruled out (Figure 2). On the other hand, several BGP_net_ values were obtained for the same water type due to the variation in the initial cell count even though the nutrient content did not change, where this phenomenon was also observed for pure bacterial cultures that reached the stationary growth phase (Fujikawa et al., 2004; Pla et al., 2015). Therefore, BGP_max_ reflects the total number of bacteria that can be maintained in water depending on the total nutrients available, part of which is utilized to maintain the initial bacteria and the remaining part is used for producing new bacteria. Only the latter part is considered when reporting BGP_net_. BGP_net_ of directly incubated samples (Prest et al., 2016a; Nescerecka et al., 2018) is relevant to predict the extent of bacterial growth that could occur in water distribution systems (Prest et al., 2016b). However, BGP_net_ may be misleading when comparing the BGP of water types that are different in initial cell count and nutrient content. For instance, the comparable BGP_net_ (50–100 × 10^3^ intact cells/mL) of directly incubated conventionally treated water (CTW) and RO-treated water (site-Remin) might indicate a similar degree of biological stability, whereas the organic nutrients were much lower in the case of site-Remin. Based on the previous discussion, reporting both BGP_max_ (after pre-treatment) and the initial cell count (before pre-treatment) may provide more information about the BGP of water: BGP_max_ is linked to the total growth-promoting nutrients, and the extent of bacterial growth during distribution can be estimated based on the difference between BGP_max_ and the initial cell count. Lastly, it could be argued that BGP_max_ does not reflect the literal meaning of the test name (i.e., BGP) because it does not include only the new cells produced (i.e., growth), but also the cells initially present in water. Therefore, adjusting the test name might be considered in the future.

### Effect of Sample Pre-treatment on BGP

Although pasteurization is used in many bioassays for assessing the biological stability of drinking water (van der Kooij and Hijnen, 1984; Sack et al., 2011; Park et al., 2016; Sousi et al., 2018), there is still a knowledge gap regarding its potential effect on the BGP of water. The comparable BGP_max_ of the pasteurized and non–pre-treated (the control) water samples (Figure 3) suggests that pasteurization does not lead to significant changes in the concentration of organic compounds available for bacterial consumption. This was observed in this study for the different types of water, i.e., conventional drinking water (CTW) containing relatively high nutrients and water produced by RO treatment (site-Remin and lab-Remin) containing ultra-low nutrients, which is in line with previous findings (Sousi et al., 2018). DOC and its fractions (characterized using LC-OCD) did not significantly change after pasteurization, even for the biodegradable part of DOC such as biopolymers and humic substances, which is in agreement with the BGP results. However, the LC–OCD characterisation could not explain the considerable increase in BGP_max_ with the other pre-treatments (i.e., autoclaving for CTW and 0.22-μm filtration for lab-Remin), where the level of biodegradable DOC remained unchanged compared with non–pre-treated samples. In addition, the weak correlation between BGP_max_ and AOC concentration can be attributed to the different nature of each test. For instance, the increase in AOC concentration in the pasteurized and autoclaved CTW was comparable despite the large difference in BGP_max_, indicating that some biodegradable compounds formed during autoclaving could not be consumed by the specific bacterial strains used in the AOC (P17/NOX) test unlike the natural bacterial communities used in the BGP bioassay (Hammes and Egli, 2005; Ross et al., 2013). Moreover, the slight increase (1 μg-C/L) in AOC concentration after pasteurising lab-Remin and site-Remin might be insignificant to be observed with the BGP test based on cell count, where low AOC concentrations did not lead to significant bacterial growth in drinking water (van der Kooij, 1992; Wang et al., 2014).

The systematic increase in BGP_max_ of the autoclaved CTW samples (75–85%) may be attributed to their relatively high nutrients, which increases the chance of nutrient denaturing, and/or to the autoclaving process that can convert the large DOC molecules to more biodegradable compounds (Servais et al., 1987). On the other hand, the considerable increase (140–320%) in BGP_max_ of ultra-low nutrient water (i.e., RO-treated water) pre-treated by 0.22-μm filtration was likely due to the leaching of biodegradable compounds from the filters (Khan and Subramania-Pillai, 2006), despite the intensive cleaning before using. The effect of filtration was exclusive for ultra-low nutrient water owing to the high sensitivity of such water even to slight leaching, whereas no measurable effect of filtration was observed for CTW that has a relatively higher nutrient content. Therefore, BGP bioassays based on pre-treatment by filtration may lead to misleading results for ultra-low nutrient water such as remineralized RO permeate, whereas those bioassays can still be applied for waters with rather high organic nutrients such as those included in the study of Farhat et al. (2018). As a result, pasteurization is a suitable pre-treatment for ultra-low nutrient water as well as conventional drinking water with a relatively higher nutrient content.

### The Ability of (remineralized) RO Permeate Bacteria to Utilize Organic Carbon

Several water types have been used for the addition of natural bacterial inocula, including activated carbon filtrate, tap water, bottled water, and raw water (Hammes and Egli, 2005; Park et al., 2016; Prest et al., 2016a; Nescerecka et al., 2018). In addition to those inoculum types, this study examined the ability of bacteria naturally present in (remineralied) RO permeate to utilize organic carbon with various molecular characteristics. The results (Figures 4, 5) clearly demonstrated that the readily available organic carbon of acetate or glucose was effectively consumed by (remineralized) RO permeate bacteria, despite the very low cell count and the potentially limited number of bacterial species (especially in lab-Remin) (Belila et al., 2016). These results indicate that (remineralized) RO permeate bacteria possess the metabolic pathways to extract energy from glucose and acetate, which are commonly present in several bacterial species (Gray et al., 1966; Berg et al., 2002; Karakashev et al., 2006). The small molecular size of glucose and acetate (belong to monosaccharides), and the possibility to convert them into energy by a broader range of bacteria may be the reasons that lab-Remin and site-Remin bacteria were able to consume it as effectively as the other bacterial inocula. The average yields considering all inocula on glucose (5.00 ± 0.65 × 10^6^ cells/μg-C) and acetate (4.45 ± 0.35 × 10^6^ cells/μg-C) were in line with the reported values (e.g., acetate, 1 × 10^6^ to 1 × 10^7^ cells/μg-C) for natural bacteria (Hammes and Egli, 2005; Prest et al., 2016a; Farhat et al., 2018) or pure strains (van der Kooij et al., 1982).

In contrast to glucose and acetate, variations in bacterial growth between the studied inocula were observed when complex organic carbon was used. Laminarin is a polysaccharide of glucose, consisting of polymers varying in the molecular size (<5,000 Da) (Percival and Ross, 1951; Smith et al., 2011). These characteristics of laminarin indicate that the indigenous RO permeate bacteria (i.e., in non-inoculated lab-Remin) lack the enzymes required for the extracellular hydrolysis of high molecular weight (HMW) polymers present in the laminarin mixture to glucose (Boraston et al., 2004; Hrmova and Fincher, 2009). Hence, they were able to grow only on the low molecular weight (LMW) polymers in the laminarin mixture. The similar yields on glucose and laminarin for all the other inocula (i.e., site-Remin, ACF, CTW, and AGW) suggests that they contain more diverse consortia that are able to completely hydrolyze laminarin to glucose and subsequently to energy molecules. The degradation of gelatin indicates the presence of extracellular enzymes (e.g., gelatinase) that are produced by certain bacterial species to liquefy gelatin (Vroman and Tighzert, 2009). However, the indigenous RO permeate bacteria (i.e., in non-inoculated lab-Remin) seem to completely lack such enzymes, as the growth was negligible compared with the other inocula. Although aerobic (Stewart and Halvorson, 1953; Shiba and Simidu, 1982; Narayan et al., 2008) and, to a lesser extent, anaerobic bacteria (Hoeffler, 1977; Whaley et al., 1982; Bruns et al., 2001) are capable of hydrolyzing gelatin, the results of this study suggest that the aerobic bacteria are more effective in degrading the complex gelatin [40,000–50,000 Da (Sack et al., 2014)] into smaller compounds (e.g., amino acids) that can be further utilized for cell maintenance and proliferation. This higher effectiveness was demonstrated in previous studies (Knowles and Smith, 1970; Tran and Unden, 1998; Trchounian et al., 1998), where aerobes obtained more energy from gelatin compared with anaerobes.

Taken together, the results suggest that BGP on the model carbon (glucose, acetate, laminarin, and gelatin) is dependent on: the natural bacterial inoculum used (i.e., the composition of bacterial communities); and the organic carbon characteristics (i.e., the complexity and molecular size). The aerobic bacterial consortia originating from treated water showed the highest yield factors for all the carbon sources. However, each carbon source may promote the growth of different aerobic bacterial species, as shown elsewhere (Sack et al., 2014) for biofilm grown in drinking water installations, where gelatin was utilized by a broader range of bacterial strains compared with polysaccharides. Therefore, further research would be needed to investigate this phenomenon for bulk water bacteria.

Regarding the bacterial growth on the natural DOC, the observed different growth for each inoculum type suggests that some bacterial strains are incapable of effectively utilizing the organic carbon present naturally in water. A similar effect was demonstrated by Joret et al. (1991), where different pure bacterial strains inoculated separately in the same water type showed considerably different abilities to convert the biodegradable DOC to new cells. Therefore, the most likely reason for the results of this study is the limited number of bacterial strains, particularly in (remineralized) RO permeate, to utilize the HMW organic compounds that form the largest proportion of the natural DOC in fresh water (Amon and Benner, 1996; Regan et al., 2017). This explanation is in line with the results discussed above, where the growth of certain bacterial communities on HMW compounds of the model carbon was limited. The BGP of indigenous RO permeate bacteria (i.e., in non-inoculated lab-Remin), that could efficiently utilize LMW compounds, on the natural DOC was only 25% of that of the aerobic ACF and CTW bacteria, that could efficiently utilize LMW and HMW compounds. This suggests that the natural DOC contains at least 75% of HMW compounds, as previously observed by Amon and Benner (1996). Furthermore, the growth on the natural DOC was equivalent to AOC concentrations of 40–70 μg-C/L (calculated using the yield obtained in this study), forming 1.5–2.7% of the natural DOC (∼2.75 mg-C/L). This ratio is consistent with previous findings for various water types (van der Kooij et al., 1989; Hu et al., 1999; Hem and Efraimsen, 2001; Shi-Hu et al., 2008; Prest et al., 2016b). Understanding the ability of bacteria to utilize HMW compounds is essential, since such compounds form a significant fraction of DOC in drinking water. Moreover, the actual bacterial growth in distribution systems was found to be strongly influenced by the concentration of HMW compounds, rather than easily biodegradable substances (Hijnen et al., 2018).

Based on the previous discussion, an aerobic bacterial inoculum originating from treated water, such as activated carbon filtrate or finished conventionally treated water, seems to effectively consume the available organic carbon resulting in reliable BGP outcomes.

### Practical Implications

The optimized BGP bioassay for ultra-low nutrient water was applied for RO-treated drinking water. The results confirmed the superior effectiveness of RO treatment in reducing the BGP_max_ of drinking water as previously reported (Park and Hu, 2010; Sousi et al., 2018). Moreover, the stable BGP_max_ over the seasons can be attributed to the small variation in nutrients of the source groundwater (Nescerecka et al., 2018). RO treatment is a promising technology to produce drinking water with a high degree of biological stability, which highlights the need for sensitive bioassays to measure the BGP.

## Conclusion

The optimized BGP bioassay for ultra-low nutrient water considers:

1. Expressing the BGP results as the maximum bacterial growth obtained during the incubation period after pre-treatment (BGP_max_). Moreover, reporting the initial cell count (before pre-treatment) together with BGP_max_ provides more information about the predicted bacterial growth during water distribution.
2. Pasteurization pre-treatment of water samples, where no effect on the BGP_max_ was observed for ultra-low nutrient water (remineralized RO permeate) and conventional drinking water. On the other hand, pre-treatment by autoclaving and 0.22-μm membrane filtration resulted in a considerable increase in BGP_max_ for some water types, where the filtration effect was exclusively observed for ultra-low nutrient water.
3. Not using (remineralized) RO permeate bacteria as inoculum, where significant limitations in their ability to utilize complex organic carbon (laminarin, gelatin, and natural DOC) were observed. Instead, aerobic bacteria of conventional drinking water should be used to ensure utilizing the majority of organic compounds, and thus, obtaining a reliable estimation of the BGP of water.

By using the optimized BGP bioassay over a 2-year period, the BGP_max_ of drinking water was significantly reduced (>75%) by applying RO-based treatment to produce ultra-low nutrient water that can limit bacterial growth.

## Data Availability Statement

All datasets generated for this study are included in the article/Supplementary Material.

## Author Contributions

MS did the experiments listed in this study, as well as the writing. The other co-authors contributed to this publication by the periodic progress meetings to give feedback. In addition, the co-authors reviewed the submission and gave their feedback to improve it.

## Conflict of Interest

The authors declare that the research was conducted in the absence of any commercial or financial relationships that could be construed as a potential conflict of interest.
