# Supplementary material for: Measuring Bacterial Growth Potential of Ultra-Low Nutrient Drinking Water Produced by Reverse Osmosis: Effect of Sample Pre-treatment and Bacterial Inoculum
Source: Front Microbiol. 2020 Apr 29;11:791. doi: 10.3389/fmicb.2020.00791 (PMC7201026; doi:10.3389/fmicb.2020.00791)
Supplement: Supplementary file 1 [file Table_1.DOCX]

Supplementary information for:

**Measuring bacterial growth potential of ultra-low nutrient drinking water produced by reverse osmosis: Effect of sample pre-treatment and bacterial inoculum**

Mohaned Sousi^a,b^, Sergio G. Salinas-Rodriguez^a^, Gang Liu^c,e*^, Jan C. Schippers^a^,
Maria D. Kennedy^a,e^, Walter van der Meer^b,d^

^a^ Department of Environmental Engineering and Water Technology, IHE Delft Institute for Water Education, Westvest 7, 2611 AX Delft, Netherlands

^b^ Faculty of Science and Technology, University of Twente, Drienerlolaan 5, 7522 NB Enschede, Netherlands

^c^ Key Laboratory of Drinking Water Science and Technology, Research Centre for Eco-Environmental Sciences, Chinese Academy of Sciences, Beijing, 100085, P.R. China

^d^ Oasen Drinkwater, Nieuwe Gouwe O.Z. 3, 2801 SB Gouda, Netherlands

^e^ Department of Water Management, Faculty of Civil Engineering and Geoscience, [Delft University of Technology](http://www.tudelft.nl/), Mekelweg 2, 2628 CD Delft, Netherlands

**Summary:** this supplementary information contains 4 figures and 1 table in 7 pages.

**Figure S1.** *The relationship between the growth in total and intact bacterial cells in lab- and site-remineralized RO permeate (lab-Remin, site-Remin, A) and conventionally treated water (CTW, B).*

| Initial cell count BGP_max_ |
| --- |
|  |

**Figure S2.** *Initial cell count and maximum bacterial growth (BGP_max_) of conventionally treated water (CTW) with different pasteurisation durations. Inoculum of 10 × 10^3^ intact cells/mL originating from the same water was added. Error bars represent the measurement variations of 3 separate tests, with triplicate vials per test (in total, n = 9). Growth rate (µ) was in the range of 0.85-1.00 for all samples.*

| Laminarin  Glucose  Acetate  Gelatin  Glucose  Laminarin  Acetate  Gelatin  Acetate  Glucose  Laminarin  Gelatin  Acetate  Glucose  Gelatin  Laminarin |
| --- |

**Figure S3.** *BGP of lab-Remin inoculated with bacteria originating from site-Remin (A), ACF (B), CTW (C), and AGW (D) at different carbon concentrations and sources. The BGP is expressed as the maximum bacterial growth during an incubation period of 20 days at 30 °C (BGP_max_). Error bars represent the measurement variations of 3 separate tests, with triplicate vials per test (in total, n = 9).*

**Table S1.** *Properties of conventionally treated water (CTW) and site-remineralised RO permeate (site-Remin).*

| **Parameter** | **CTW** | **site-Remin** |
| --- | --- | --- |
| T (°C) | 11.8 | 12.7 |
| pH | 8.1 | 7.8 |
| Turbidity (FTE) | <0.1–0.4 | <0.1–0.3 |
| O_2_ (mg O_2_/L) | 8.3 | 9.1 |
| SI | -0.07–0.18 | -4.38–0.23 |
| Total hardness (mmol/L) | 1.2 | 1.1 |
| Cond. (mS/m) | 63.225 | 20.94063 |
| Bicarbonate (mg HCO_3_/L) | 219.5 | 132.2 |
| Cl (mg Cl/L) | 74.6 | n.a. |
| F (mg F/L) | 0.1 | <0.02 |
| Al (µg Al/L) | 3.8 | n.a. |
| SO_4_ (mg SO_4_/L) | 50.7 | n.a. |
| Na (mg Na/L) | 101.6 | n.a. |
| Fe (mg Fe/L) | 0.01 | n.a. |
| Mg (mg Mg/L) | 15.5 | 3.5 |
| Mn (mg Mn/L) | <0,005 | <0,005 |
| Ca (mg Ca/L) | 22.7 | 40.4 |
| DOC (mg C/L) | 6.4 | <0.2 |
| NH_4_ (mg N/L) | <0.02 | <0.02 |
| NO_2_ (mg N/L) | <0.003 | <0.003 |
| NO_3_ (mg N/L) | 2.89 | 0.23 |

n.a. not available

**Table S2.** *BGP, AOC, and organic carbon fractions (with LC–OCD) of lab-remineralized RO permeate (lab-Remin), site-remineralized RO permeate (site-Remin) and conventionally treated water (CTW) with different pre-treatments.*

|  | | **BGP (× 10^3^ intact cells/mL)** | **AOC  (P17, NOX) (µg-C/L)** | **LC–OCD (µg-C/L)^*^** | | | | | | |
| --- | --- | --- | --- | --- | --- | --- | --- | --- | --- | --- |
|  |  |  |  | **TOC** | **DOC** | **Biopolymers** | **Humic substances** | **Building blocks** | **Neutrals** | **Acids** |
| **lab-Remin** | No pre-treatment | 40 ± 5 | 3.8 ± 0.2 | <200 | <200 | <100 | <200 | <200 | <200 | <200 |
|  | Pasteurisation at 70 °C for 30 min | 41 ± 8 | 5.4 ± 0.1 | <200 | <200 | <100 | <200 | <200 | <200 | <200 |
|  | Autoclaving at 121 °C for 15 min | N/A | N/A | N/A | N/A | N/A | N/A | N/A | N/A | N/A |
|  | 0.22 µm filtration | 180 ± 60 | 7.4 ± 1.0 | <200 | <200 | <100 | <200 | <200 | <200 | <200 |
|  | | | | | | | | | | |
| **site-Remin** | No pre-treatment | 94 ± 5 | 1.9 ± 0.1 | <200 | <200 | <100 | <200 | <200 | <200 | <200 |
|  | Pasteurisation at 70 °C for 30 min | 98 ± 2 | 2.9 ± 0.0 | <200 | <200 | <100 | <200 | <200 | <200 | <200 |
|  | Autoclaving at 121 °C for 15 min | N/A | N/A | N/A | N/A | N/A | N/A | N/A | N/A | N/A |
|  | 0.22 µm filtration | N/A | N/A | N/A | N/A | N/A | N/A | N/A | N/A | N/A |
|  | | | | | | | | | | |
| **CTW** | No pre-treatment | 677 ± 60 | 4.2 ± 0.3 | 6000 | 5980 | <100 | 4260 | 969 | 764 | <200 |
|  | Pasteurisation at 70 °C for 30 min | 610 ± 25 | 12.5 ± 1.8 | 5870 | 5880 | <100 | 4250 | 972 | 746 | <200 |
|  | Autoclaving at 121 °C for 15 min | 1,180 ± 160 | 10.7 ± 0.7 | 5900 | 5870 | <100 | 4270 | 1000 | 723 | <200 |
|  | 0.22 µm filtration | 655 ± 40 | N/A | N/A | N/A | N/A | N/A | N/A | N/A | N/A |

* Limit of detection for TOC, DOC, humic substances, building blocks, neutrals and acids is 200 µg-C/L, biopolymers is 100 µg-C/L, and AOC is 1 µg-C/L; N/A, not measured
